# Macro and meso level influences on distributed integrated COPD care delivery: a social network perspective

**DOI:** 10.1186/s12913-021-06532-y

**Published:** 2021-05-23

**Authors:** Wendy Hartford, Sevinj Asgarova, Graham MacDonald, Mary Berger, Sayra Cristancho, Laura Nimmon

**Affiliations:** 1 Department of Occupational Science and Occupational Therapy, Faculty of Medicine, University of British Columbia, 2211 Wesbrook Mall T325, BC V6T 2B5 Vancouver, Canada; 2 University of British Columbia, Vancouver, Canada; 3 Dalhousie University, Halifax, Canada; 4 Centre for Education Research and Innovation, Schulich School of Medicine and Dentistry, University of Western Ontario Canada, Medical Sciences Building, Suite 100, N6G 2V4 London, Ontario Canada; 5 Centre for Health Education Scholarship, Faculty of Medicine, University of British Columbia, 429-2194, Health Sciences Mall, V6T 1Z3 Vancouver, Canada

**Keywords:** Chronic obstructive pulmonary disease, Integrated care, Distributed healthcare, Social networks, Family physician, Respirologist, Respiratory therapist

## Abstract

**Background:**

Care guidelines for people with chronic obstructive pulmonary disease (COPD) recommend an integrated approach for holistic, flexible, and tailored interventions. Continuity of care is also emphasised. However, many patients with COPD experience fragmented care. Discontinuities in healthcare and related social services are likely to result in disjointed rather than integrated care which can negatively affect patient health outcomes. The purpose of this qualitative study was to improve our understanding of, and how, contextual features pertaining to structures and processes of COPD integrated care influence delivery of care within patients’ healthcare networks.

**Methods:**

We conducted individual interviews with 28 participants (9 patients, 16 healthcare professionals, and 3 spousal caregivers). Participants were recruited through the lung clinic at a city hospital in western Canada. We employed a social network paradigm to analyse and interpret the data.

**Results:**

The analysis revealed an overarching theme of fragmented COPD care with two sub-themes: (1) Funding shortfalls and availability of resources, and (2) Dis(mis)connected communication pathways. The overarching theme depicts variations, delays, and discontinuities in patient care. The sub-themes describe how macro level influences and meso level shortfalls were perceived to influence the availability of respiratory care resources that contributed to fragmented COPD care.

**Conclusions:**

Employing a social network lens drew particular attention to family physicians’ pivotal role in delivering community-based COPD care. While an integrated approach to care is recommended by care guidelines, institutional and organizational structures and processes, such as financial and communication structures, may inhibit delivery of integrated care. Thus, macro and meso level structures and processes have the potential to shape patient care by constraining family physicians’ purposive and communication actions necessary for facilitating an integrated distributed approach to care. We propose a context of care which fosters a context for family physicians’ delivery of patient-centered care. Integrated care delivery may improve patients’ wellbeing and alleviate financial constraints on the healthcare system.

## Background

Chronic obstructive pulmonary disease (COPD) is a global [1] and local [2, 3] health burden, and a major cause of disability and death [2, 3]. People with COPD often have comorbidities such as asthma, diabetes, and hypertension [4] which increases both health and economic burden [2, 4]. The needs of people with COPD appear to be well-established [5] and care guidelines (Table 1) commonly recommend an integrated care approach for the treatment and management of COPD [4, 6].

**Table 1:** Guidelines for COPD care

Overall guidelines for best practice for an integrated comprehensive approach to COPD care include: diagnosis of COPD confirmed with spirometry, clinical evaluation, and comprehensive management to include pharmacological, non-pharmacological, and educational interventions [4, 6].
Goals: prevent disease progression, relieve symptoms, improve exercise tolerance and health status, reduce mortality, and prevent and treat complications and exacerbations.
Four components 1. Assess and monitor disease 2. Reduce risk factors: such as tobacco use 3. Manage stable COPD: based on severity of disease, pharmacological, non-pharmacological, and health education 4. Manage exacerbations: common causes infection and air pollution require pharmacological treatment and non-invasive ventilation.

There is no clear definition of the term “integrated care” [7]. The term can be used, for example, to describe managed care, continuity of care, patient-centered care, and or integrated delivery systems [7, 8]. Integrated care also considers organizational and professional aspects of a healthcare delivery system [8]. In relation to patient care, integrated care more often relates to collaboration between various healthcare practices, such as family physician, respiratory specialist, therapist, pharmaceutical, and pulmonary rehabilitation services, that are necessary to treat and manage COPD [7, 9]. Integrated care with these collaborative characteristics has been shown to improve patient health outcomes, particularly with respect to decreased exacerbations and hospitalization [11, 12]. At the organizational level, integrated care refers to the organizations and services that deliver healthcare, such as health authorities and private service providers, and is thought to reduce healthcare costs [9–11].

Central to concepts of integrated care are notions of holistic care, patient education and self-management [9], flexibility and continuity [13] and tailored interventions. These are considered critical features which adapt to changes in patients’ needs over the course of the disease [14]. Importantly, addressing social isolation that many patients experience, due to physical and psychological distress, should be a prominent consideration of integrated care [13, 15]. Support structures and processes, such as protected resources [16], spirometry testing [6], appropriate transition mechanisms [9], and flexible referral procedures [13], are also requirements for integrated care. Structures that support healthcare provider/patient relationship development should also be present [10].

Despite recommendations for integrated COPD care, studies suggest that many patients experience fragmented COPD care [6, 18]. For instance, Wodskou and colleagues [13] found that while patients were not dissatisfied with their overall care, patients’ needs contrasted with resource availability leading to dissimilar access to services and fragmented care (e.g., referral times and availability of rehabilitation programs). Furthermore, current guidelines for integrated COPD management, such as patient education and managing exacerbations, may not be widely implemented [9]. Some speculate that this may, in part, be due to varying perceptions of integrated care between different stakeholders (patients, their healthcare team members, and health organizations), and across and within different care settings [8,13,17]. In addition, a diversity of care settings, and healthcare structures and processes suggest that a universal model of integrated COPD care is unlikely and fraught with challenges [7]. At a broader level, discontinuities in healthcare and related social services can result in fragmented rather than integrated care [8].

Barton et al. [15] suggest that a social network approach to COPD patient care may provide a deeper understanding of organizational and individual structures and processes which support patient care. Moreover, others have suggested that primary care perspectives of the inter-relations that exist between clinical (micro), professional and organizational (meso), and system (macro) levels of care may improve our understanding of integrated care requirements [8].

A social network approach focuses on the patterns of social relations that develop between individuals and groups [19]. Individual contacts, connections, and group attachments provide the basis for the creation of relational data which merge and enable organization of social action [19], such as COPD care. This approach also aims to explore structural properties of networks and how they influence social action [19]. Relations that are generated between individuals and groups are not attributed to individuals, but are considered properties of the connections between individuals and groups [19].

According to the fundamental premise of a social network approach, the relations, and the patterns they form construct social life [20]. This perspective is distinct from research approaches which focus on individual and attribute-based contexts [20]. In relation to integrated COPD care, a social network approach brings attention to the ways in which relational connections form between actors involved in COPD care which influence the social action of delivery of care. Thus, we assume individuals in a network are connected and focus on how resources and communication are mobilized, or not, through network relational connections. This differs from research approaches which interview multiple stakeholders, but primarily consider the professional attributes individual actors bring to COPD care delivery as discrete entities.

However, research concerning the dimensions of patients’ healthcare social networks is limited. Presently, network structures, such as the composition of patients’ networks, and processes which support an integrated approach to care are not well understood in the context of COPD care. As a result, we lack insight into the ways contextual meso and macro level network practices shape patients’ health outcomes particularly where care is provided in distributed community settings rather than in a single location. Using a social network lens, we define community as “people providing support and services to those to whom they are connected” rather than a geographic location with supports and services ([20], p.19). As such, while care services may be geographically distributed, our premise is that communities of distributed integrated care can be constructed.

Without this knowledge we are at risk of perpetuating fragmented care that in turn may lead to poor patient outcomes and wasted resources [8, 16, 17].

The purpose of this study is to improve our understanding of, and how, contextual features pertaining to structures and processes of integrated care influence delivery of COPD care within patients’ healthcare networks. We aimed to broaden the perspective of COPD treatment and management, and build on previous research on patients’ experiences and perspectives of integrated COPD care [13, 18]. We anticipate that a social network lens may expand our understanding of patients’, caregivers’, and healthcare professionals’ experiences and provide nuanced interpretations of care practices [13, 21]. Our goal is to identify structures and processes to support promoting distributed integrated COPD care, and ultimately high-quality patient healthcare by answering the following questions: 1). What are the healthcare network structures and processes which contribute to distributed integrated COPD care? 2). What are the challenges of delivering distributed integrated COPD care?

## Methods

### Study design

This qualitative study is a secondary data analysis [22, 23] of a larger study which investigated how COPD teams (people with COPD and their healthcare networks) negotiate and make decisions about a patient care plan. In this secondary analysis we specifically study the influence of healthcare network structures and processes on distributed integrated COPD care. The questions that we explore in this study were not part of the parent study, but emerged during the primary analysis [22, 23]. This secondary data analysis considers a subset of data from the parent study [22, 23]: patients receiving care in the community.

### Social network paradigm

A social network paradigm stresses the importance of group members’ “perceptions and experiences of the context in which they act” and in the construction of its social meaning ([19], p. 15). Particular attention is given to characteristics that enable and/or constrain choice and agency [19, 24, 25]. Within the conceptual landscape of social network analysis, communication (flow of information) and purposive action (flow of resources) merge forming a foundation for network cooperation [19]. Within social networks, sets of ties link various actors and allow for or block the flow of information and resources between actors [24]. In the present analysis, we define patients’ healthcare networks as being egocentric. An egocentric network is based on patients, their selected healthcare providers, and the structures and processes in which interactions occur in the context of COPD care. Egocentric social networks are not isolated from other social networks [24, 26]. For instance, patients’ COPD healthcare networks are interwoven with organisational (e.g., healthcare professional organizations) and institutional networks (e.g., healthcare authorities). Organization and institutional networks frame egocentric social networks [19]. A social network perspective may sharpen insights into network structures and processes that shape distributed integrated COPD care.

### Data Collection

The research team for both the parent study and this secondary analysis included the principal investigator LN, a senior investigator SC, a research coordinator SA, and three research assistants GM, MB and WH. For the parent study we used a purposive sampling strategy to recruit COPD patients and members of their healthcare networks in a two phased process. Patients were recruited through the hospital lung clinic in a large urban area in western Canada. SA was invited to visit the clinic to describe the study and provide consent packages to interested patients and caregivers. We conducted semi-structured, in-depth interviews with all participants which lasted between 30 and 90 min. SA conducted face-to-face interviews with all patient participants. During the interviews, each patient was asked to name at least 3 members of their healthcare network [27]. With patients’ permission, SA contacted and invited patients’ healthcare network members to participate in the study. These participants were interviewed separately by phone or in person by SA. In total, for the parent study, we recruited 10 patients, 19 healthcare providers, and 3 spousal caregivers for a total sample of 32 participants. All interviews were audio recorded and SA also provided field notes. The interview protocol was designed to promote discussion around healthcare network members and their role in providing patient care, communication, and relationships with other network members. Patients were identified as team (network) members in the parent study and the interview questions were posed to all participants. For example, “When a problem comes up how do you communicate with the other team members?” When, patients were asked “Who is on your healthcare team?”, healthcare team members were asked “Who is on the patient’s healthcare team?” The recorded interviews were transcribed verbatim, and anonymized. For this secondary data analysis, we drew on participant data from 9 patients, their 16 healthcare providers, and three spousal caregivers for a total sample of 28 participants. The data from one patient and their team was excluded as this patient received institutionalised care in a long-term care facility rather than in the community.

### Data Analysis

Regular debriefing meetings, with the original research team, were conducted to undertake this qualitative secondary data analysis. In accordance with a social network lens, we focused on identifying ties between actors within patients’ healthcare networks and how actors (patients and their healthcare providers) were connected. Since ties represent pathways of communication between actors [19, 24], we sought to identify communication modes (e.g., electronic communication systems, face-to-face conversation) and how information and resources (healthcare services) flowed between actors. Patterns of communication, information, and resource flow were of significant interest in relation to how they constrained or enabled integrated care.

The analytic process followed a three-stage iterative and cyclical systematic process: item analysis, pattern analysis and structural analysis. Item analysis involved compiling groups of similar items of interest (e.g., different healthcare services patients used) which led to the identification of primary codes for organising the data [28]. Pattern analysis involved a process of comparison, contrast, and integration which led to organizing items together in higher order patterns. For instance, patterns were identified around patients’ experience accessing care and of continuity of care. Structural analysis involved bringing together pieces of an analytic puzzle to create an overall structural insight of the phenomena under exploration [28]. During structural analysis, we incorporated many initial codes and developed theoretically informed themes that drew on social network theory [19, 24]. This conceptually driven stage of analysis elucidated structures and processes that influenced integrated care. NVivo (version 11 Pro) was used to organize and manage the data. To provide participant anonymity, pseudonyms have been applied.

Ethics approval for this study was received from the Behavioral Research Ethics Board of the university. Written consent was obtained from all participants prior to the individual interviews.

## Results

The composition of patients’ COPD healthcare networks was diverse as was gender, age, and marital and socioeconomic status (Table 2). All networks except two included a family physician and respirologist. Patients’ networks varied with respect to respiratory therapists, nurse clinician, pharmacists, spousal caregivers, and other specialists. Patients received care through three different health authorities responsible for delivering care in the area. Patients were served by hospital respiratory clinics, independent community physician clinics, and specialist managed clinics. For the majority of patients, family physicians rather than respirologists, were the primary COPD care provider. Overall patients appeared to be satisfied with their care, but were less satisfied with organizational processes, referrals, and consultation times which led to discontinuities in care.

**Table 2:** Characterisation of patients and their COPD healthcare networks: Legend: PWD = people with disabilities; U = universal healthcare insurance; EH = extended healthcare insurance

Patient	Age/gender	COPD Diagnosis	Healthcare network (not interviewed)	Other health issues	Marital status/ (children)	Employment (status)	Annual income (health benefits)
**Case 1**	58/female	2013	Family practitioner, respiratory therapist/educator, clinical COPD research coordinator, and respirologist.	Lung tumor	Single/ (none)	Artist/ (retired)	$14,000/ (PWD)
**Case 2**	75/female	~ 2009	Pharmacist and respirologist, (family practitioner, nephrologist)	Kidney function cataract surgery	Divorced/ (2)	Social worker/ (parttime)	100,000/ (U, EH)
**Case 3**	75/male	2009	Spousal caregiver, pharmacist, and respiratory therapist/educator	Cancer remission	Married/ (4)	Communication specialist/ (retired)	$75,000/ (U, EH)
**Case 4**	75/male	2004	Family practitioner and respirologist	NA	Widower/ (2)	Smelter/ (retired)	$120,000/ (U)
**Case 5**	78/male	2009	Nurse clinician and respirologist (family practitioner)	NA	Divorced/ (2)	Visual art/ (retired)	$18,000/ (U, AL)
**Case 6**	73/male	2018	Spousal caregiver and movement disorder neurologist (respirologist, family practitioner, vascular surgeon)	Parkinson’s disease	Married/ (2)	Mechanic/ (retired)	$30,000/ (U)
**Case 7**	58/male	~ 2009	Respiratory therapist (family practitioner, nurse clinician)	Hepatitis HIV	Single/ (none)	Labourer/ (retired)	$19,000/ (PWD)
**Case 8**	70/female	2011	Family practitioner, pharmacist, and respirologist	Glaucoma	Divorced/ (2)	NA/ (retired)	$18,000/ (U)
**Case 9**	75/female	2001	Spousal caregiver, nurse clinician, respirologist, and family physician	NA	Married/ (2)	NA/ (retired)	NA/ (U)

The analysis revealed an overarching theme of fragmented COPD care, with two interwoven subthemes: (1) Funding shortfalls and availability of resources, and (2) Dis(mis)connected communication pathways. The first theme introduces the overarching concept of fragmented flow of resources. The two subthemes then describe how macro and meso level influences and shortfalls were perceived to influence the availability of respiratory care resources that contributed to fragmented COPD care.

### Fragmented COPD care

Participants framed COPD care as currently “piecemeal” (*respiratory therapist 3)* while acknowledging the benefits of integrated care and healthcare moving in that direction. Patients experienced changes in healthcare providers, and given stretched resources, it was rare for independent physician community clinics to take a multidisciplinary approach:

*But otherwise in the physician’s clinics it’s not a multidisciplinary approach. I think they go in and see the physician and that’s it, and then if they need social work they’re referred back out… within the clinic of a specialist there isn’t other disciplines working or part of a team…. (respiratory therapist 2).*

Patients referred to their family physicians as the person or “traffic cop” (patient 2) who directed their care.

 While the majority of patients had been referred to respirologists, not all patients were referred to respiratory clinics, or respiratory therapists/educators resulting in dissimilar patterns of care:

*[P]atients are going to be referred to a specialist based on the severity of their condition, because a lot of family physicians will try to manage the condition on their own without putting that additional burden on the specialist system. Not all patients are going to be referred to a respiratory therapist. Clinic E is a very unusual situation where they have their own respiratory therapist…I think patients for the most part have their family physician treating them (nurse clinician).*

The referral process, which required patients to renew their family physician’s respirologist referral after 6 months, delayed and disrupted continuity of care.

*So from the time we first ask Dr. Anderson [family physician] to go to a COPD clinic, to getting to see Dr. Oliver [respirologist], that was a year and a half. And then from seeing Dr. Oliver to when we’ll see him again after Ryan [patient] has, you know, his C.T. scan and everything, that’s going to be another five or six months. So now we’re, like, two years in (spousal caregiver 2).*

Differing health professional perspectives of care had the potential to interfere with patient care plans. Despite guidelines for treatment and management family physicians may not always choose to follow respirologists’ recommended care plans: *“But there’s always some doctors [i.e., FP] who have their own opinion about what is best for the patients…”* (*respirologist 1*).

Participants indicated that funding shortfalls contributed to these discontinuities in COPD care.

### Funding shortfalls and availability of resources

The major resource for funding COPD care was provincial universal health insurance plan which provided for all health services deemed medically necessary. Increased financial support appeared to be essential to provide adequate health professionals and resources to meet the needs of the COPD community. Hospital administrative decisions regarding bed availability could result in patients being discharged before they were ready and/or before community care had been arranged.

*I feel like it’s a combination of the way our system is structured. I think there is limited capacity sometimes, and I think there might be financial…it’s not incentives. That’s not the right word. But there are other factors that influence some of the decisions being made (family physician 2).*

It seemed that funding COPD care may depend on how well healthcare administrators understood the cost and health benefits associated with providing recommended COPD care, but *“they don’t always either have the resources or the ability to come up with those decisions” (respirologist 1)*. Insufficient budgetary support for COPD care also had a significant influence on shortfalls in care. In particular, shortfalls in the physician payment structure (mostly fee-for-service) were perceived to limit family physician capacity to manage integrated COPD care. Family physicians were perceived as being overloaded with patients and paperwork, and consequently had insufficient time to spend time with complex patients. Against these shortfalls, there were inferences of unnecessary consultations and medical tests: *“But what I see is some of these investigations are ordered annually, routinely. And I don’t think it’s actually supported by much evidence “(family physician 2).*

Furthermore, the healthcare system could not support specialist care for every COPD patient. Resources to assist family physicians were available in the form of rapid telephonic access to consultative expertise and various care plan tools on the government website. However, family physicians were generally not well equipped for diagnosing and managing COPD patients.

*…the vast majority of COPD patients are taken care of by primary care physicians…the downside of primary care is they have limited time to spend with the patient. And because the patient may have heart failure and diabetes…they’ve multiple issues to be dealt with. So most G.P.’s [family physicians] don’t have access to an educator. They don’t have access to spirometry…they can’t track their patients (respirologist 2)*.

In addition, there were insufficient family physicians, clinic nurses, community respiratory therapists/educators, and other allied health professionals to meet the therapeutic and educational needs of individual COPD healthcare networks. The availability of respiratory therapists varied between health authorities: “*In Health Authority C I think they have a team of, like, 12 or 13 of them actually…. there’s only two of us that work in the community in Main Site A*” *(respiratory therapist 3)*. In particular, adequate numbers of respiratory therapists were perceived to have the potential of reducing specialist referral wait list. Moreover, although transition teams managed patient transition back to the community, patient follow-up was limited and may not be continued over the long term:

*So at the time of discharge I would have a note saying that the transition team, they’ll arrange to see her after she’s discharged…I don’t think she actually is continuously followed by this transition team. I think it’s really just when she gets discharged home… (family physician 4)*.

There were indications that behaviour change for smoking cessation required long term support: “*we’ve been working pretty consistently on that too for several years*” *(family physician 1).*

 One respiratory therapist referred to a smoking cessation program and a COPD support group that they had set up outside of the clinic, but these did not appear to be common features of COPD care due to lack of resources. Patients appreciated the opportunity to attend rehabilitation programs with several patients benefitting from re-enrollment:

*…went through the six weeks and did their exercise and listened to the lecture. But the second time going through it was much better than the first time… And then when you’re going to these groups you’re talking to other people that have the same problem…I learned an awful lot from that (patient 3).*

However, re-enrollment was not typically available. In addition to funding shortfalls and resource availability, communication flow through networks also contributed to fragmented care.

### Dis(mis)connected communication pathways

 Participants emphasised the benefits of a community wide electronic communication system. Current communication modes often left one of the care providers out of the “loop” at the expense of patient care. Healthcare providers experienced difficulties in communication due to dissimilar communication systems used by different health authorities, hospitals, and physician/specialist clinics. Patient electronic medical records (EMRs) were not available across different health authority networks and clinics. Reports and referrals were mailed to clinical offices outside of a particular system, faxes and phone modes often required follow-up communication, and network meetings were mostly impractical due to geographical location and healthcare provider availability. These disconnected communication modes resulted in delays in receiving reports and disrupted patient care.

*Mainly not getting information, that’s a big challenge. So if I never got a consult note. If I didn’t get a discharge from hospital. Some information about what the next step is. If it’s a handwritten note, let’s say, from the emergency department that I can’t read…Typically once I get a note there are fairly clear recommendations on what the outcome was and what the next steps should be (family physician 4)*.

Different perspectives of patient care contributed to a lack of a unified approach. This manifested in divergent patient care plans and disjointedness in care which appeared to be exacerbated by inadequate communication modes. For instance, processes, such as hospital discharge following emergency care of exacerbations, may be influenced by organizational perspectives and formulary, and hospital ways of doing things. Moreover, these processes appeared to be disconnected from community ways of living. Lack of communication with the patient’s family physician could result in a hospital discharge plan than did not take the patient’s life context into account. As family physician 1 explained *’the actual circumstance for the client isn’t always visible to them [hospitalists]*’, but ‘there’s no point’ for hospitalists to prescribe the hospital regimen of inhaler medication because their patient was, ‘*not going to do four times a day inhaler treatment…. So she’s on a once daily inhaler, and it’s a compassionate supply. And it’s worked out really well for her.’*

Despite a common prescription recording system for healthcare professionals, delays occurred when clarifying prescriptions and obtaining prescription refills. A common prescription notation eased physician/pharmacist communication when used correctly. However, there was no commonly accepted communication mode between pharmacists and physicians for resolving prescription queries and renewals.

*So there are different offices, different policies, different doctors, different policies. And they’re all allowed to do that, so there is not basically a single way to communicate…nothing on the College of Physicians basically by law to say, okay, if you want to communicate and have a refill, you have to fax the doctor and doctor’s supposed to answer your fax. No. There’s no such thing… (pharmacist 3)*.

Some family physicians renewed patient prescriptions at the request (phone or fax) of a pharmacist, while others insisted that the patient schedule a regular appointment. All of these communication modes often fostered delays and contributed to discontinuity of care.

Overall, it seemed that shortfalls in, communication, funding, and resources for COPD care diminished patients’ and caregivers’ experiences of care and also attenuated healthcare providers’ ability to deliver collaborative and integrated COPD care.

## Discussion

Our social network informed insights enrich our understanding of the structures and processes which contribute to an integrated approach to distributed community COPD care: integration of geographically distributed services. The structure of a network is shaped by the formation of connections between individuals and groups, and influences social action [20], in this instance delivery of COPD care. It is this structure which constrains or enables agency of network actors and which determines network dynamics and effectiveness [19]. Through a social network lens, our findings illuminate areas of vulnerability in COPD networks which detract from network effectiveness. Significantly, fragmented flow of resources contributed to an overall perception of fragmented COPD care.

Patient healthcare networks were diverse. For the most part patient participants appeared to be satisfied with the care provided by their healthcare network members. At the same time, participants’ perceptions of limited availability of resources, driven by funding shortfalls, contributed to an overarching sense of fragmented care. Fragmented care was further influenced by an apparent lack of a unified care network approach to COPD care. This was aggravated by unequal distribution of pulmonary rehabilitation resources across the region. In particular, disparate communication modes inhibited information transfer and an integrated approach to care.

Our findings draw particular attention to the pivotal role of family physicians providing for patients in a distributed COPD community; an expectation that is reported in the literature [8, 29]. Nonetheless, our findings revealed that limited consultation time, work overload, and lack of resources restricted family physicians’ ability to provide an integrated approach to care. There is also an assumption present in the literature that family physicians are familiar with and follow provincial and professional care guidelines (Table 1) for an integrated approach to care [31]. However, our findings imply that this may not be the case. Furthermore, our findings suggest that organizational (meso level) and institutional level (macro level) structures and processes which control resources inhibit an integrated approach to COPD care. In particular, inadequacies in financial and communication structures may shape patient care by inhibiting purposive action and communication, such as the transfer of resources and information, resulting in disjointed and asymmetrical delivery of resources.

### Financial Structures

The emphasis on the need for increased financial support to provide resources for community care dominated the findings. Notably, improvements to financial structures which supported increased access to and availability of respirologists and respiratory therapists were identified as crucial to patient care management. In particular, improving access to specialists, decreasing specialist consultation wait times, and providing adequate respiratory therapists across health authorities were highlighted by participants as significant structural changes that could improve access to care. Furthermore, our insights, which support the literature [6, 13, 30] suggest family physicians may not have the time, or access to resources, to adequately address the complex needs of COPD patients and support patient self-management. People with COPD require respiratory therapy, smoking cessation education [31], and anxiety management [32]. Furthermore, COPD often has to be managed along with other complex co-morbidities [1, 4, 6]. Self-management education, which includes pulmonary rehabilitation, is a key component of COPD care, and has been associated with decreased hospital admissions due to exacerbations [1, 2, 6]. Our findings resonate with the literature which suggest changes to organizational (e.g., financial) structures that allow family physicians time for addressing the needs of complex patients [10], along with access to essential COPD resources required to implement guidelines [30], may better support integrated care.

### Communication structures

Insights from our findings also suggest, as reported in the literature [14, 33, 34], that an effective community communication system is a critical support for integrated distributed chronic illness care. Communication pathways that support information flow between network members are essential linkages within social networks [19, 24]. These linkages refer to both individual network member communication behaviour/characteristics and network structures which promote or inhibit transfer of information [24]. In particular, the rate and reliability of network decisions can be affected not only by geographic distance, but also by varied communication structures (e.g., electronic medical records) [24]. The varied disconnected communication modes used by different community members in our study contributed to delays and discontinuity of care. This was particularly evident for patient transfer from hospital to community, and potentially resulted in a fragmented approach to care. A common communication system that links healthcare professionals would enable flow and sharing of information and knowledge around patients’ needs and improve decision making [35]. A shared communication system may also expedite referrals and report sharing, and reduce clinical office management costs [34, 35]. Despite these potential benefits of shared communication systems, the uptake of a unified electronic communication system appears to be elusive. As identified in our study and the literature, clinical locale (e.g. hospitals and clinics) [10, 33, 34] use a variety of medical record systems. Moreover, medical services (e.g., drug and diagnostic information) [36] also use different electronic systems. These electronic systems are often incompatible: a considerable organizational barrier to integrated communication. Differences in information organization of various clinical groups along with hierarchical structures that are invariably present also inhibit integrated communication [33].

In summary, our findings suggest that family physicians may not have access to the resources necessary for purposive action and communication which facilitate an integrated approach to distributed COPD care. This may lead to fragmented patient care, poor patient health outcomes, increased healthcare related costs, and increased overall societal burden of care [8]. Thus, the interplay of macro and meso level structures and processes have the potential to shape patient care.

### Implications

Our analysis suggests that existing guidelines for an integrated approach to COPD care are perhaps idealistic, and may not be attainable within distributed community care. The limited application of COPD guideline-based care in distributed settings and the need to redesign care delivery have been identified in the literature [9, 16]. Our study insights highlight the pivotal role of family physicians in care delivery, but at the same time draw attention to how financial and communication structures potentially restrict an integrated approach to care. An integrated approach to care is expected to improve patient health outcomes and reduce economic costs associated with treatment [11, 12]. However, without adequate resource funding [18] and integrated communication [33] positive aspects of integrated care may never materialise.

We propose a concept of care that is specific to distributed community care and which facilitates family physicians’ delivery of patient-centered care. In accordance with a social network paradigm [19], this concept of care affords specific attention to organizational and institutional structures which support family physicians’ purposive action and communication: that is, the provision of financial and communication structures that facilitate the transfer of resources (pharmacological and non-pharmacological) and information between patient healthcare network members to enhance patient care. Individual networks are not isolated from other personal or organizational networks [19]. Thus, it is important to recognise that distributed care should not be dissociated from hospital care, or social culture practices and policies [37]. Communications structures, both technological and human, between these networks are needed to provide fully integrated care. Future research may provide insights as to how these structures interact. Finally, our findings give rise to questioning whether family physicians face an increased burden of care when resources are not available. An area for future research could explore family physicians experience of increased burden of care, and how institutional and organizational structures and processes could be redesigned to improve family physician accessibility to resources.

### Strengths and limitations

The strength of this secondary data analysis was accessibility to the parent study data and the original research team who worked closely together through all stages of this subset data analysis [38]. In addition, from a social network perspective, the research question was a good fit with the parent study research question [38], particularly as ego-centered networks and organizational structures are interrelated [19]. However, as this data set was confined by the parent study, data saturation may not have been possible as the researchers were unable to ask participants’ additional questions that emerged from this analysis. Insights from this study express similarities to findings from other Canadian [6, 14] and international studies [10, 13, 18]. While this study’s findings are context specific, and not broadly transferable, they may provide valuable insights for similar COPD population groups.

At the time of data collection (2019), limited uptake of telemedicine as a resource for improving access to COPD care [14] restricted the interview protocol to investigation of in-person consultations. However, the advent of COVID-19 pandemic has increased the use of telemedicine [39]. Telemedicine may possibly enhance integrated care [14, 16] and is an area for future research. Finally, we were unable to recruit all healthcare network members identified by patients. Thus, our analysis may not reflect the experiences of complete networks. In addition, this analysis focused only on the organizational and structural components of healthcare networks. We recognize the importance of network member interactions, power relations, and hierarchical structures shaping participating health professionals’ collaborations. These insights are to be reported separately by our research team. We also recognize the importance of interactions between different healthcare networks. Some patients mentioned other medical conditions during their interviews. The data was not sufficient to draw any conclusions regarding how other co-occurring disease management networks interfaced with and/or contributed to COPD care. Exploring how co-occurring disease management networks interface is an important area for future search which can contribute to understanding the complexities of chronic disease management. Despite these limitations, this secondary data analysis has identified gaps in organizational structures which provide opportunities for further exploration and research. For instance, given that many people with COPD experience hospitalization due to exacerbations and disease progression [2, 6]) identifying structures and processes that support institutional COPD care is an important area for future research.

## Conclusions

We explored the experiences of people with COPD and their community healthcare networks to gain a broader and deeper understanding of the healthcare network structures and processes which contribute to distributed integrated COPD care. We also explored the challenges of delivering integrated care. We identified diverse patient healthcare networks, and the influence of macro level (broader healthcare system, and meso level (organizational)) structures and shortfalls. Through a social network lens our findings highlighted family physicians’ pivotal role in delivering community-based COPD care. Our insights emphasize that while an integrated approach to care is recommended by care guidelines, institutional and organizational structures and processes, such as financial and communication structures, may inhibit delivery of integrated care. Moreover, macro and meso level structures and processes have the potential to shape patient care by constraining family physicians’ purposive and communication actions necessary for facilitating an integrated distributed approach to care. We propose a context of care that is specific to distributed integrated care and which is supported by macro and meso level structures which facilitate family physicians’ delivery of patient-centered care. Improving efficacy of care has the potential to improve patients’ wellbeing and alleviate healthcare system financial constraints.

## Data Availability

The datasets generated and analysed during the current study are not publicly available due to the sensitive and confidential nature of the raw data on which the conclusions of the manuscript rely. This study does not have ethics approval to share the participants’’ narratives publicly. The data sets are available from the corresponding author on reasonable request.

## Abbreviations

COPD: Chronic obstructive pulmonary disease
LN: Laura Nimmon
SC: Sayra Cristancho
SA: Sevinj Asgarova
GM: Graham MacDonald
MB: Mary Berger
WH: Wendy Hartford
